# Takayasu arteritis presenting as cerebral aneurysms in an 18 month old: A case report

**DOI:** 10.1186/1546-0096-6-4

**Published:** 2008-01-31

**Authors:** Pamela F Weiss, Diana A Corao, Avrum N Pollock, Terri H Finkel, Sabrina E Smith

**Affiliations:** 1 Division of Rheumatology, Department of Pediatrics, The Children's Hospital of Philadelphia, University of Pennsylvania School of Medicine, Philadelphia, PA, USA; 2 Department of Pathology, The Children's Hospital of Philadelphia, University of Pennsylvania School of Medicine, Philadelphia, PA, USA; 3 Department of Radiology, The Children's Hospital of Philadelphia, University of Pennsylvania School of Medicine, Philadelphia, PA, USA; 4 Division of Neurology, Department of Pediatrics, The Children's Hospital of Philadelphia, University of Pennsylvania School of Medicine, Philadelphia, PA, USA

## Abstract

**Background:**

Central nervous system involvement occurs in as many as twenty percent of Takayasu arteritis cases. When central nervous system disease is present, it typically manifests as cerebral ischemia or stroke. There are rare reports of intracranial aneurysms in adults with Takayasu arteritis, but none in children.

**Case presentation:**

We describe a case of Takayasu arteritis in an 18 month old girl who presented with a ruptured cerebral aneurysm. Full body magnetic resonance angiography revealed bilateral iliac, pelvic and intragluteal aneurysms, irregular terminal aorta, and stenotic renal arteries. Iliac vessel biopsy showed a lymphocytic infiltrate and giant cells localized to the internal elastica.

**Conclusion:**

This case highlights cerebral aneurysm as a highly unusual initial manifestation of Takayasu arteritis and demonstrates the challenges of diagnosis, treatment, and assessment of response to therapy in TA in children.

## Background

Takayasu arteritis (TA) is a large vessel systemic granulomatous vasculitis primarily involving the aorta and its major branches. TA is the third most common childhood vasculitis worldwide, but it is relatively rare in North America and accounts for 2% of all childhood vasculitis in the United States[1,2]. The majority of pediatric patients with TA present during adolescence [3]. Using the American College of Rheumatology (ACR) TA classification criteria, the presence of 3 of the 6 following features is diagnostic: (1) age of onset before 40 years, (2) claudication, (3) decreased brachial artery pulse, (4) blood pressure difference of >10 mm Hg between the arms, (5) bruit over the subclavian arteries or aorta and (6) arteriogram abnormality [4]. According to the more recent EULAR (European League Against Rheumatism)/PReS (Paediatric Rheumatology European Society) consensus criteria, diagnosis of TA requires angiographic abnormalities (conventional, CT, or MRI/A) of the aorta or one of its major branches plus one or more of the following: (1) claudication or decreased peripheral artery pulses, (2) blood pressure difference >10 mm Hg, (3) bruits of the aorta or its major branches, (4) hypertension [5]. However, diagnosis is challenging in the early inflammatory phase when only non-specific symptoms are present and imaging is non-diagnostic. Twenty percent of TA patients have central nervous system (CNS) involvement, usually manifesting as cerebral ischemia or stroke [6,7]. There are 19 reports of intracranial aneurysms in TA, but none in individuals younger than 15 years [8-11]. Ringleb *et al. *reported that middle cerebral artery (MCA) involvement was most common [9].

The gold standard for TA imaging is angiography. However, it is invasive and cannot detect thickened vessel walls, an early sign of inflammation. MRI/A and PET scan can detect luminal diameter changes and disparities in vessel metabolic activity, respectively, indicative of early inflammation [12,13]. Once the diagnosis of TA is made, treatment is challenging. Corticosteroids may achieve remission rates of 60%, although more than half of these patients flare with tapering [6]. There are case reports of treatment with methotrexate, azathioprine, infliximab [14,15] and adalimumab [16]. Cyclophosphamide is reserved for recalcitrant cases or severe, systemic involvement [17].

## Case Presentation

**Figure 1:**
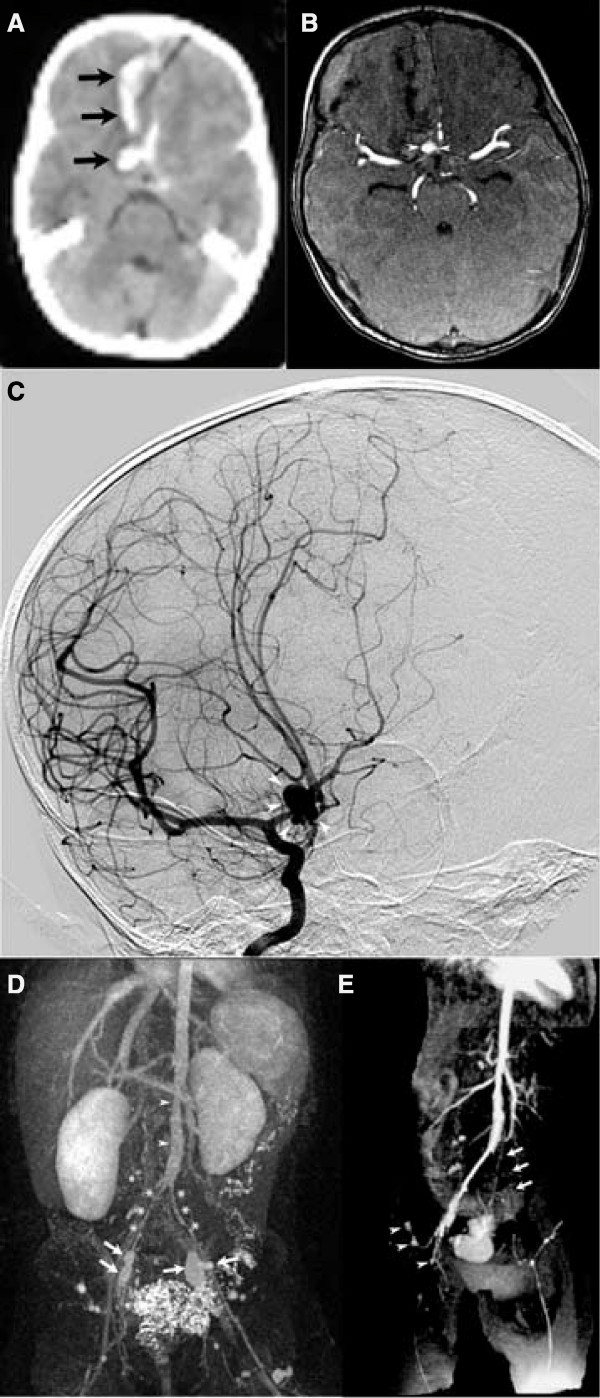
Non-contrast head CT (Fig 1A) demonstrates frontal lobe hemorrhage (arrows). Source image from MRA of the brain (Fig 1B) demonstrates 8 mm aneurysm in the distal A1 segment of the right ACA (arrowheads). DSA (AP/oblique) from right ICA injections (Fig 1C) confirms aneurysm of the distal A1 ACA segment. 3-D TOF MRA abdomen (Fig 1D) with bilateral internal iliac artery aneurysms (arrows), narrowing of mid-abdominal aorta (arrowheads), diffusely narrowed bilateral common and external iliac arteries (asterisks). 2-D TOF MRA abdomen (Fig 1E) shows worsening involvement of left common iliac artery (arrows), with right external and internal iliac arterial aneurysms unchanged (arrowheads).

An 18-month old Caucasian girl presented to our hospital following a generalized tonic-clonic seizure. On examination she was unresponsive, hypotonic, and mechanically ventilated. She was normotensive and afebrile, and there were no audible bruits on examination. Significant laboratories included the following: white count 32,400/μl, hemoglobin 9.6 g/dl, platelet count 342,000/μl. Normal laboratories included the following: PT, PTT, and complete metabolic panel. Erythrocyte sedimentation rate (ESR) and C-reactive protein (CRP) were not drawn. Past medical history was significant for failure to thrive requiring supplemental tube feeding. Family history was notable for an aunt with an asymptomatic cerebral aneurysm. Head CT (Fig 1A) demonstrated hemorrhage and dilated ventricles. Brain MRI/A demonstrated aneurysms of the right anterior cerebral artery (ACA) (Fig 1B) and the left internal carotid artery (ICA), confirmed by digital subtraction angiogram (DSA; Fig 1C). The ACA aneurysm was clipped and a ventriculostomy was placed. Her course was complicated by seizures and vasospasm, causing ischemic strokes in bilateral ACA, subcortical middle cerebral artery (MCA) and posterior cerebral artery (PCA) territories, as documented by MRI.

**Figure 2:**
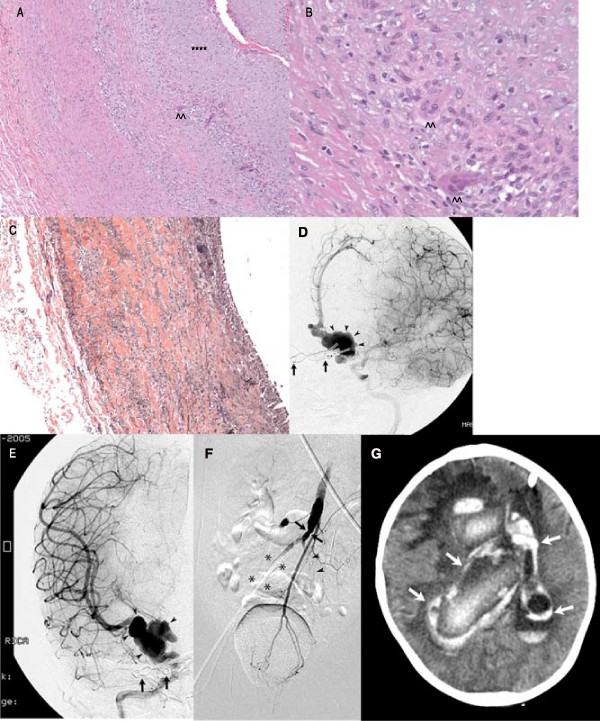
H&E stain (200×, Fig 2A; 400×, Fig 2B) from right internal iliac artery shows transmural vasculitis with intimal thickening (asterisks) and giant cells (arrowheads). Elastic stain reveals disruption of elastic lamina (Fig 2C). AP DSA, fusiform aneurysms supraclinoid left ICA (arrowheads; Fig 2D), right ICA (arrowheads; Fig 2E). Note aneurysm clips (arrows). DSA, diminutive, irregular aorta (arrows; Fig 2F). Right common iliac artery not identified (asterisks), no flow distal left common iliac artery (arrowheads). Non-contrast head CT (Fig 2G) demonstrates acute hemorrhage (arrows).

Three weeks later, she developed vomiting and irritability. Significant laboratories included white count 9,500/μl, hemoglobin 9.7 g/dl, platelet count 737,000/μl, ESR 37 mm/hr, and CRP 2.7 mg/dl. Head CT showed a new hemorrhage, and angiography showed recurrence of the clipped ACA aneurysm. Full body MRA revealed iliac artery aneurysms, aortic narrowing (Fig 1D), and gluteal aneurysms. Blood pressure remained normal and there were no audible bruits. At this point, suspicion for a genetic vasculopathy was high and systemic vasculitis was not suspected. Repeat imaging showed progression in the left common iliac artery (Fig 1E) and narrowing of the renal arteries. A hip effusion was noted. Renal ultrasound and blood pressure were normal. Laboratory studies included a normal chromosomal variant (46, XX, inv(9) (p13q21)), and normal elastin gene and JAG1 locus. Collagen synthesis, sequencing of TGFBR2, urine copper and serum ceruloplasmin were normal. The left iliac artery aneurysm was repaired; pathology showed non-specific changes of elastic lamina disruption and chronic inflammatory infiltrate. At discharge 3 months later, she had spastic quadriparesis but was sitting with assistance. Five months after presentation, the right iliac artery aneurysm was repaired. Pathology demonstrated a transmural vasculitis with intimal thickening, giant cells (Fig 2A, B) and elastic lamina disruption (Fig 2C). Despite the presence of giant cells, suspicion for a genetic vasculopathy remained high.

Seven months after presentation, she was found vomiting, posturing and unresponsive. Head CT showed new intraventricular hemorrhage, and DSA revealed fusiform aneurysms in the supraclinoid ICA's (Fig 2D,E), right lumbar, and internal maxillary arteries. Brachial artery pulses were not measurable due to line placement; her hands were cyanotic, radial and posterior tibial pulses were undetectable, and her feet were cool. White count was elevated 17,500/μl, hemoglobin was 10.5 g/dl, platelets were 189,000/μl, CRP was elevated (4.7 mg/dl) and ESR was normal (2 mm/hr). Intravascular stent placement and coil embolization of the right cerebral aneurysm were performed. After re-review of the biopsies and imaging, TA was finally suspected and treatment with pulse solumedrol and cyclophosphamide was initiated. Two days later she had a new intraventricular hemorrhage and the left ACA aneurysm was coiled. On imaging, her aorta was narrowed (Fig 2F). Despite aggressive management, she had a massive intracerebral hemorrhage (Fig 2G). The family agreed to discontinue medical support given her poor prognosis.

**Figure 3:**
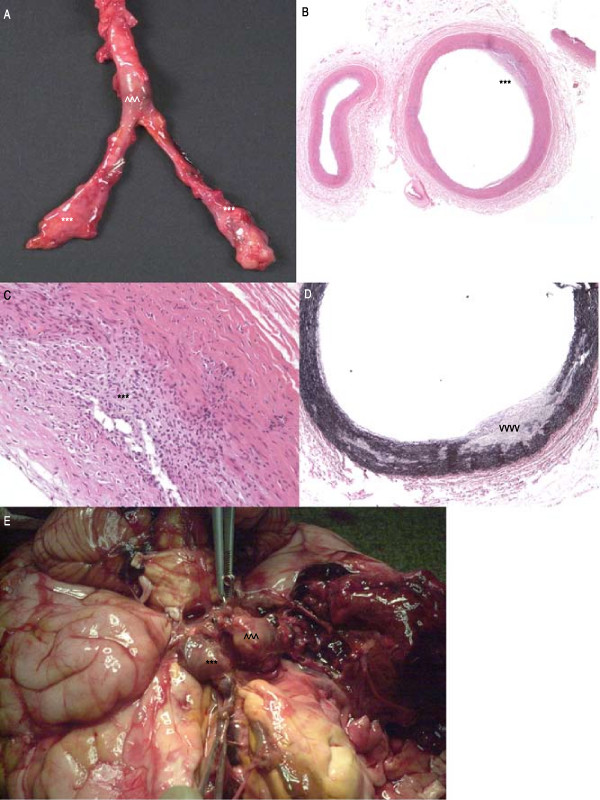
Gross photograph, aortic and bilateral iliac artery aneurysms (Fig 3A). Left subclavian artery, focal intimal thickening (asterisks; H&E stain 40×, Fig 3B), transmural lymphohistiocytic infiltrate (asterisks; H&E 200×, Fig 3C), elastic lamina disruption (arrowheads; elastic stain 40×, Fig 3D). Gross photograph brain, saccular aneurysms right ICA (arrowhead), ACoA (asterisks; Fig 3E).

Autopsy showed fusiform aneurysms of the descending aorta with thrombosis of 95% of the luminal cavity (Fig 3A). The rest of the aorta was grossly unremarkable, although histologic examination showed transmural lymphohistiocytic vasculitis with rare giant cells in the subclavian artery (Figs 3B–D), abdominal aorta (above the aneurysm), and iliac arteries. There was intimal thickening and proliferation in the common carotids and left anterior descending coronary arteries. Cerebral vessels had saccular aneurysms in the right ICA and anterior communicating artery (ACoA; Fig 3E). There was massive intracerebral hemorrhage with frontal lobe necrosis and intraventricular rupture. Microscopic examination of the cerebral vessels showed chronic inflammation and rare giant cells. The involvement of large and medium arteries, the histology and radiology were distinctive for TA.

## Conclusion

This patient did not fulfill the ACR criteria for TA, which were primarily designed for adult TA detection [4]. She did, however, fulfill the revised EULAR/PReS criteria for TA [5], given (1) radiographic narrowing of the aorta and renal arteries, and (2) undetectable radial and tibial pulses. Her presentation highlights the diagnostic challenges in a young child since, unfortunately, TA was not considered until late in her clinical course. Our patient presented with her first aneurysm at 18 months. Signs of inflammation were, however, present at 10 months, manifesting as failure to thrive. This case demonstrates that TA can cause cerebral aneurysms in children and raises the following question: should all patients with cerebral aneurysms be evaluated for systemic aneurysms and/or vasculitis?

The differential diagnosis of cerebral aneurysms in children is large and includes genetic, infectious and inflammatory causes. Systemic non-inflammatory vasculopathies associated with cerebral abnormalities include fibromuscular dysplasia, polycystic kidney disease, Rendu-Osler-Weber disease, and moyamoya syndrome. Genetic associations include Ehlers-Danlos, tuberous sclerosis, neurofibromatosis, Seckle and Alagille syndromes. Infectious etiologies are HIV, mycotic infections, tuberculous meningitis, and CNS Lyme disease. Systemic inflammatory vasculitides such as TA, Kawasaki disease, Behçet's, primary angiitis of the central nervous system, and polyarteritis nodosa are unusual etiologies of cerebral aneurysms. In this case, the evaluation initially centered upon genetic and non-inflammatory etiologies. As the case evolved, the underlying diagnosis was determined pathologically. When multiple cerebral aneurysms are present, screening for systemic vasculopathy should be considered. If systemic aneurysms are found, as in this case, an extensive investigation is warranted and should include evaluation for inflammatory vasculopathies.

Once diagnosis of TA is made, assessing response to treatment is challenging. Prior studies have shown clinical signs and symptoms of disease and elevated acute phase reactants are poorly correlated with disease activity. In the presence of hardware or clips from aneurysmal repair, non-invasive studies such as MRA and CTA are limited due to artifact. One recent report discussed the utility of non-invasive measurement of aortic arterial elastic properties with M-mode echocardiographic images in children with TA [18]. Furthermore, the optimal interval for serial imaging during remission is uncertain.

This case highlights cerebral aneurysm as an initial manifestation of systemic vasculitis and demonstrates the challenges of diagnosis, treatment, and assessment of response to therapy in TA in children.

## List of abbreviations

TA, Takayasu arteritis, MRA, magnetic resonance angiography, CNS, central nervous system, MCA, middle cerebral artery, ACA, anterior cerebral artery, ACoA, anterior communicating artery, DSA, digital subtraction angiogram, ICA, internal carotid artery, MRI, magnetic resonance imaging, CT, computed tomography, PCA, posterior cerebral artery, ESR, erythrocyte sedimentation rate, CRP, C-reactive protein, TOF, time of flight

## Competing interests

The author(s) declare that they have no competing interests.

## Authors' contributions

PFW and SES were involved in patient care and in drafting the manuscript. DC helped draft the pathology-related portion of the manuscript and created the pathology figures. ANP drafted all neuroradiology figures and helped revise the radiology portion of the manuscript. THF was involved in patient care and in revising the manuscript critically for intellectual content.
